# Detection of HOCl-driven degradation of the pericardium scaffolds by label-free multiphoton fluorescence lifetime imaging

**DOI:** 10.1038/s41598-022-14138-5

**Published:** 2022-06-20

**Authors:** B. P. Yakimov, I. I. Vlasova, Y. M. Efremov, E. G. Maksimov, E. A. Shirshin, V. E. Kagan, P. S. Timashev

**Affiliations:** 1 World-Class Research Center “Digital Biodesign and Personalized Healthcare”, Sechenov First Moscow State Medical University, Trubetskaya 8, Moscow, Russia 119048; 2 Faculty of Physics, M.V. Lomonosov Moscow State University, 1-2 Leninskie Gory, Moscow, Russia 119991; 3 Department of Advanced Biomaterials, Institute for Regenerative Medicine, Sechenov First Moscow State Medical University, Trubetskaya 8, Moscow, Russia 119048; 4 Faculty of Biology, M.V. Lomonosov Moscow State University, 1-12 Leninskie Gory, Moscow, Russia 119991; 5 Center for Free Radical and Antioxidant Health, Department of Environmental and Occupational Health, University of Pittsburgh, Pittsburgh, PA 15261 USA; 6 Faculty of Chemistry, M.V. Lomonosov Moscow State University, 1-3 Leninskie Gory, Moscow, Russia 119991

**Keywords:** Imaging and sensing, Fluorescence spectroscopy, Biomedical materials

## Abstract

Artificial biomaterials can significantly increase the rate of tissue regeneration. However, implantation of scaffolds leads not only to accelerated tissue healing but also to an immune response of the organism, which results in the degradation of the biomaterial. The synergy of the immune response and scaffold degradation processes largely determines the efficiency of tissue regeneration. Still, methods suitable for fast, accurate and non-invasive characterization of the degradation degree of biomaterial are highly demandable. Here we show the possibility of monitoring the degradation of decellularized bovine pericardium scaffolds under conditions mimicking the immune response and oxidation processes using multiphoton tomography combined with fluorescence lifetime imaging (MPT-FLIM). We found that the fluorescence lifetimes of genipin-induced cross-links in collagen and oxidation products of collagen are prominent markers of oxidative degradation of scaffolds. This was verified in model experiments, where the oxidation was induced with hypochlorous acid or by exposure to activated neutrophils. The fluorescence decay parameters also correlated with the changes of micromechanical properties of the scaffolds as assessed using atomic force microscopy (AFM). Our results suggest that FLIM can be used for quantitative assessments of the properties and degradation of the scaffolds essential for the wound healing processes in vivo.

## Introduction

In tissue engineering and regenerative medicine, scaffolds provide mechanical support and biochemical signals to ensure cell survival and differentiation^1^. Decellularization of animal tissues and organs allows to fabricate extracellular matrix (ECM) that maintains the mechanical and biochemical properties of the native ECM and constitutes an ideal niche for tissue regeneration^2,3^. The biomaterial stimulates cell recruitment and tissue remodeling, and at the same time, it gradually degrades. The rate and mechanisms of scaffold degradation largely determine the immune response to scaffold implantation and wound healing efficiency.

Decellularized bovine pericardium (DBP) is of particular interest in medicine due to its availability, biocompatibility, and tunable physicochemical properties^4^. DBP is a cheap and robust material widely used in cardiac surgery, orthopedics, general and pulmonary surgery, and dentistry^5^. Cross-linking of DBP by chemical agents like glutaraldehyde, epoxy-compounds, and genipin modulates immunogenicity of the biomaterial, improves its proteolytic stability and prolongs its degradation^4,6^. However, undue delay of the scaffold degradation may result in the excessive inflammatory response and foreign body formation. Therefore, design of the new cross-linking agents and optimization of the scaffold architecture and degradation is a hot spot in regenerative medicine^7^.

Implantation of scaffolds initiates the immune response whereby neutrophils are the first immune cells recruited and activated at the site of tissue damage^8,9^. Neutrophil activation is accompanied by the secretion of proteins (e.g., collagenase, gelatinase) of intracellular granules capable of degrading the biomaterials. In addition, activated neutrophils release myeloperoxidase (MPO) which—in the presence of H_2_O_2_﻿—generates hypochlorous acid (HOCl). HOCl is a powerful oxidizing agent that can oxidize and degrade not only biological molecules but also carbon-based nanomaterials^10–12^.

Physical techniques, such as atomic force microscopy (AFM), have been successfully employed to evaluate the changes in the structural properties and degradation of scaffolds^13–16^. Optical methods also allow for rapid quantitative assessment of the physicochemical properties of the biomaterials as well as for probing the metabolic status of the surrounding cells. Particularly promising is the application of the multiphoton fluorescence tomography combined with fluorescence lifetime imaging (MPT-FLIM), which permits to visualize the deeper tissue layers with molecular-specific contrast^17^. The MPT-FLIM has been previously used to characterize the degradation of collagen-based scaffolds^18–23^. FLIM combined with Raman spectroscopy also allows to assess the degree of cross-linking in collagen-containing pericardium scaffolds using the fluorescence signal of the genipin-induced cross-links^19^. Multispectral FLIM was used to monitor the enzymatic collagen degradation in bovine pericardium scaffolds^20^. Fluorescence stereomicroscopy and multiphoton tomography were used in conjunction with immunohistochemical assays and microtomography to gain insights into the degradation of DBP ex vivo^21^. The fiber-optic FLIM was applied for tracking the dynamics of the recellularization of the scaffold surface by monitoring the changes of the fluorescence lifetime^22^. Parameters of the autofluorescence lifetime of collagen oxidation products and cross-links can also be used to characterize natural collagens and their degradation in vivo^24–27^. Yet, the modification of the collagen biomaterials induced by the immune cells has not been assessed by optical techniques.

In this work, we explored the analytical power of the MPT-FLIM in evaluating the degradation of DBP scaffolds caused by simulating the neutrophils-induced oxidation. The DBP scaffolds treated with two cross-linkers, genipin and ethylene glycol diglycidyl ether (EGDE), were used as test objects.

We demonstrated that HOCl-driven degradation of pericardium scaffolds can be monitored using fluorescence lifetime response from the genipin-induced cross-links and autofluorescence of the scaffold proteins’ oxidation products. The observed fluorescence decay parameters correlated with micromechanical properties of the scaffolds measured by AFM. The developed fluorescence-based approach was tested using scaffolds degradation under the conditions simulating an immune response of human neutrophils. Our results confirm that the fluorescence decay parameters are sensitive markers for monitoring the degradation of biological scaffolds and immune responses elicited by the scaffolds.

## Materials and methods

### Sample preparation

DBP was produced as described earlier^4^ and sterilized with gamma radiation. Samples of the scaffold for NaOCl treatment were prepared under sterile conditions. Pieces of DBP weighing 10–18 mg were placed in water for a day (10 ml of water changed 2 times), then for 2–3 h in 10 ml of PBS. Finally, samples were put into PBS with 50 mM Na-phosphate buffer (pH 7.3–7.4) and treated with hypochlorite. Control samples (without treatment) were prepared in the same way two days before measurements and kept at 4–6 °C.

NaOCl was purchased from SigmaAldrich Co. as solution of 10–15% chlorine and NaOH in water (#425044). Concentration of NaOCl in stock solution was determined by measuring the absorbance at 290 nm (ε = 350 M^−1^ cm^−1^) after successive dilutions of reagent in 5 mM NaOH. The concentration was 1.7 M. Micro-aliqoutes of 0.4–1 µl of NaOCl were added to the scaffold placed into 4 ml of buffer (PBS + 50 mM NaH_2_PO_4_, pH 7.3–7.4) to reach the final concentration of 170–425 μM NaOCl. NaOCl was added once or twice a day for 5–10 days depending on the desired degree of DBP modification. The largest amount of added hypochlorite did not exceed 15 μl, while the pH of the solution changed by no more than 0.15 units. An addition of NaOCl was made when NaOCl concentration in a sample did not exceed 50 μM. The residual amount of hypochlorite was determined using the method based on the oxidation of 5-thio-2-nitrobenzoic acid TNB^28^. The total amount of NaOCl added was quantified as µmol NaOCl per mg of the scaffold. At the end of incubation, the samples were put into water for FLIM or into PBS for AFM measurements for several hours (7–8 ml changed twice).

### Neutrophils isolation and incubation with scaffold

In accordance with protocols approved by the Ministry of Health of the Russian Federation, fresh venous blood was collected from healthy donors in blue top vacutainers with 3.8% sodium citrate as an anticoagulant. Human neutrophils were isolated by a procedure utilizing Histopaque 1.077 (Sigma, St. Louis, MO, USA) as described in^29^. Human blood was mixed with 6% dextran T-500 (Sigma, St. Louis, MO, USA) in PBS at the 5:1 ratio, and erythrocytes were allowed to sediment for 30 min at room temperature (RT). The leukocyte-rich plasma was layered on top of 3 ml Histopaque (Sigma, St. Louis, MO, USA) and subjected to centrifugation (400 g, 30 min, RT). Contaminating erythrocytes were removed by hypotonic lysis with cold H_2_O. Neutrophils were washed twice with PBS (4 °C); the obtained cells were suspended in Hanks' Balanced Salt Solution (HBSS) without Ca^2+^, Mg^2+^ and with 5% of autologous serum at the concentration of 10–12 million cells per ml and stored at 4 °C before use. The cell suspension was dimidiated for two successive incubations. Neutrophils were isolated twice a day, so four incubations were performed.

DBP cross-linked with genipin (DBP-G) was treated by H_2_O and PBS as described above. A 5×5 mm DBP-G sample was placed into 0.7–0.8 ml of neutrophil suspension, to which calcium (1 mM) and magnesium (0.5 mM) were added. A control sample of scaffold was placed into HBSS, containing 5% serum, Ca^2+^ (1 mM) and Mg^2+^ (0.5 mM), followed by the addition of 25–30 μl of PBS (which is equal to the volume of reagents added to the samples with neutrophils). Neutrophil activator phorbol 12-myristate 13-acetate (PMA) was added in 15 min at the concentration of 150 nM. In 1.5 h after the beginning of incubation 100 µM H_2_O_2_ was added 3 times (with 30 min intervals) at the concentration of 100 µM. The samples were mixed by gentle pipetting every 30 or 90 min (last incubation). Three incubations were carried out at 37 °C for 3–3.5 h; the fourth incubation was performed overnight (6–7 h) at RT. Between incubations, the scaffold was washed three times with PBS. At the end of incubations, scaffold was washed with PBS and 0.2% SDS within 5–7 min to remove attached neutrophil remnants; the control scaffold was treated in the same way. Finally, the samples were kept in water several hours before FLIM measurements.

All donors had given written informed consent. A positive vote has been obtained from the ethics committee of the Sechenov First Moscow State Medical University and the experiments were in accordance with the principles of the declaration of Helsinki as revised in 2013 and protocols approved by the Ministry of Health of the Russian Federation.

### MPT-FLIM

FLIM with multiphoton excitation was performed using a custom-built multiphoton multimodal microscopy setup. Femtosecond optical parametric oscillator TOPOL-1050-C (Avesta, Russia), providing excitation in the 680–1000 nm range, was used as an excitation source. The pulse width of the exciting radiation was ~150 fs; the repetition rate was 80 MHz, average power at the excitation wavelength on the sample was 1 mW. Scanning over the sample was performed using the DSC-120 scan head (Becker&Hickl, Germany). Imaging was performed using air Plan Apochromat 20× objective with NA = 0.75. Fluorescence decay curves were detected using hybrid GaAsP detector HPM-100-40 (Becker&Hickl, Germany) with sensitivity in the 250–720 nm range and instrument response function with the time width of 50 ps. To cut off the excitation light, the 680 nm short-pass dichroic filter was used. Since the spectral characteristics of the FLIM detection system did not permit to completely eliminate the signal of the second harmonic from the collagen of scaffolds, we additionally verify the lack of ultrafast component characteristic for second harmonic signal in the decay curves both for DBP-G samples excited at 800 nm and DBP-EGDE samples excited at 730 nm. A detailed description of this procedure is presented in Supplementary Note 1.

To select the FLIM-imaging area, white light wide-field microscopy was used. Since the samples had a homogeneous structure, there were no additional requirements for the selection of image areas. The scanning depth was set to 10–20 μm from the sample surface; in this position, the fluorescence signal had the maximum amplitude.

Fluorescence decay curves were fitted using SPCImage 8.3 software (Becker&Hickl, Germany) after spatial binning (bin size was equal to 3 for DBP-scaffolds cross-linked with genipin and 10 for the processing of DBP-scaffolds cross-linked with ethylene glycol diglycidyl ether) using biexponential decay model (Eq. 1):

1 $$I\left( t \right) = \mathop \smallint \limits_{{ - { }\infty }}^{\infty } IRF\left( {t - t^{{\prime }} } \right)\left( {{\text{a}}_{1} \exp \left( { - {{{t^{{\prime }} }} \!\mathord{\left/ {\vphantom {{t^{{\prime }} } {\tau_{1} }}}\right.\kern-\nulldelimiterspace} \!\lower0.7ex\hbox{${\tau_{1} }$}}} \right) + {\text{a}}_{2} \exp \left( { - {{{t^{{\prime }} }} \!\mathord{\left/ {\vphantom {{t^{{\prime }} } {\tau_{2} }}}\right.\kern-\nulldelimiterspace} \!\lower0.7ex\hbox{${\tau_{2} }$}}} \right)} \right)dt^{{\prime }} ,$$

where $I\left( t \right)$ is the detected fluorescence signal, $IRF\left( t \right)$ is the instrument response function of the detection system, a_1_,a_2_ are the characteristic amplitudes, τ_1_, τ_2_-characteristic lifetimes of the fluorescence decay, estimated from the fit. The average fluorescence lifetime (τ_m_) was calculated as a weighted sum of fluorescence lifetimes τ_m_ = (a_1_τ_1_ + a_2_ τ_2_)/(a_1_ + a_2_). Phasor plot of data was calculated according to Digman et al.^30^, the time window of the phasor plot was chosen to be T = 4 ns, i.e. frequency of the phasor plot was equal to Ω = 2 π/4 ns^−1^ for better data representation in the phasor diagram.

The analysis of the distribution of the fluorescence decay parameters, calculation of phasor plots and data visualization were performed using custom-written Python 3 scripts using NumPy, Pandas, Matplotlib and scikit-learn libraries^31^.

To quantitatively estimate the observed bimodality in the distributions of the mean fluorescence lifetime τ_m_ obtained for DPB-G scaffolds treated with NaOCl, the fluorescence lifetime distributions were fitted using the Gaussian mixture model (scikit-learn Python library). The distributions of the mean lifetime τ_m_ for individual samples treated with 0.1, 0.25 and 0.75 μmol (NaOCl) /mg (scaffold) were fitted as a mixture of two Gaussian distributions with different means (τ_m_^(1)^, τ_m_^(2)^) and variance parameters (σ^(1)^, σ^(2)^), while the distributions of the τ_m_ parameter for the control sample and sample, treated with 1.5 μmol (NaOCl)/mg (scaffold) that did not exhibit pronounced bimodality were fitted using single Gaussian distribution. The fractions of the oxidized material were estimated as the sum of the pixels attributed to the cluster with a longer fluorescence lifetime normalized to the overall number of pixels in the distribution.

### Atomic force microscopy

AFM experiments were performed on a Bioscope Resolve AFM (Bruker, Santa Barbara, CA) system mounted on an Axio Observer inverted optical microscope (Carl Zeiss, Germany). The ScanAsyst-Fluid cantilevers (Bruker) were used with the nominal spring constant of 0.7 N/m and the nominal tip radius of 20 nm. The exact values were determined by the thermal tune method and by scanning the titanium roughness sample (Bruker), respectively. Since, like most biological samples, DBP possesses substantial mechanical heterogeneity, care was taken to obtain control and treated sample pieces from close locations on the source DBP. The samples for the AFM were cut in half and glued on the bottom of a Petri dish with different sides facing up, so both sides of the sample were accessible. The side with lower roughness and heterogeneity was selected for further analysis^4^. All AFM measurements were performed in phosphate-buffered saline at room temperature. Force Volume Mapping was conducted over 10 × 10 µm areas with 32 × 32 force curve arrays; 5–6 maps per sample were recorded. The vertical piezo movement speed was 61 µm/s, and the trigger force was ≈25 nN. Each force curve was corrected for hydrodynamic drag according to Efremov et al.^32^ and analyzed to extract Young’s modulus value using the Hertz model (Eq. 2):

2 $$F = \frac{4}{3}\frac{{\text{E}}}{{1 - \nu^{2} }}\sqrt R \delta^{\frac{3}{2}} ,$$

where *F* is the measured force, *E* is Young’s modulus, ν is the Poisson ratio (assumed to be 0.5), *R* is the tip radius, and *δ* is the indentation depth.

The AFM imaging was performed at the same conditions using the same cantilevers in Peak-Force Tapping mode. 10 × 10 µm scans with 256 × 256 points were acquired at 1 kHz oscillation frequency and 150 nm amplitude with a set point of 2 nN. The acquired topography images were processed (flattened) with Gwyddion software^33^.

### Statistics

The figures represent the results of a typical experiment out of three independent experiments. The data are shown as distribution histograms or superplots^34^ unless otherwise specified in the text.

## Results

### DBP-scaffolds degradation: fluorescence of the cross-linker

DBP-G scaffolds were treated with NaOCl, a major product of the neutrophil-driven immune response, and examined by MPT-FLIM with excitation at 800 nm and emission decay detection in the 350–680 nm range. Both in control (untreated) and oxidized (treated) samples intense fluorescent response well described by the biexponential decay model was observed. Both short and long decay components were attributed to the fluorescence signal, as characteristic lifetimes τ_1_ and τ_2_ of the decay components were significantly larger than the instrumental response function, thus excluding the influence of the second harmonic signal generated by collagen fibers (detailed description is given in Supplementary Note 1). The presence of such an intense fluorescence signal can be explained by the formation of genipin-induced cross-links in collagen^35^.

**Figure 1:**
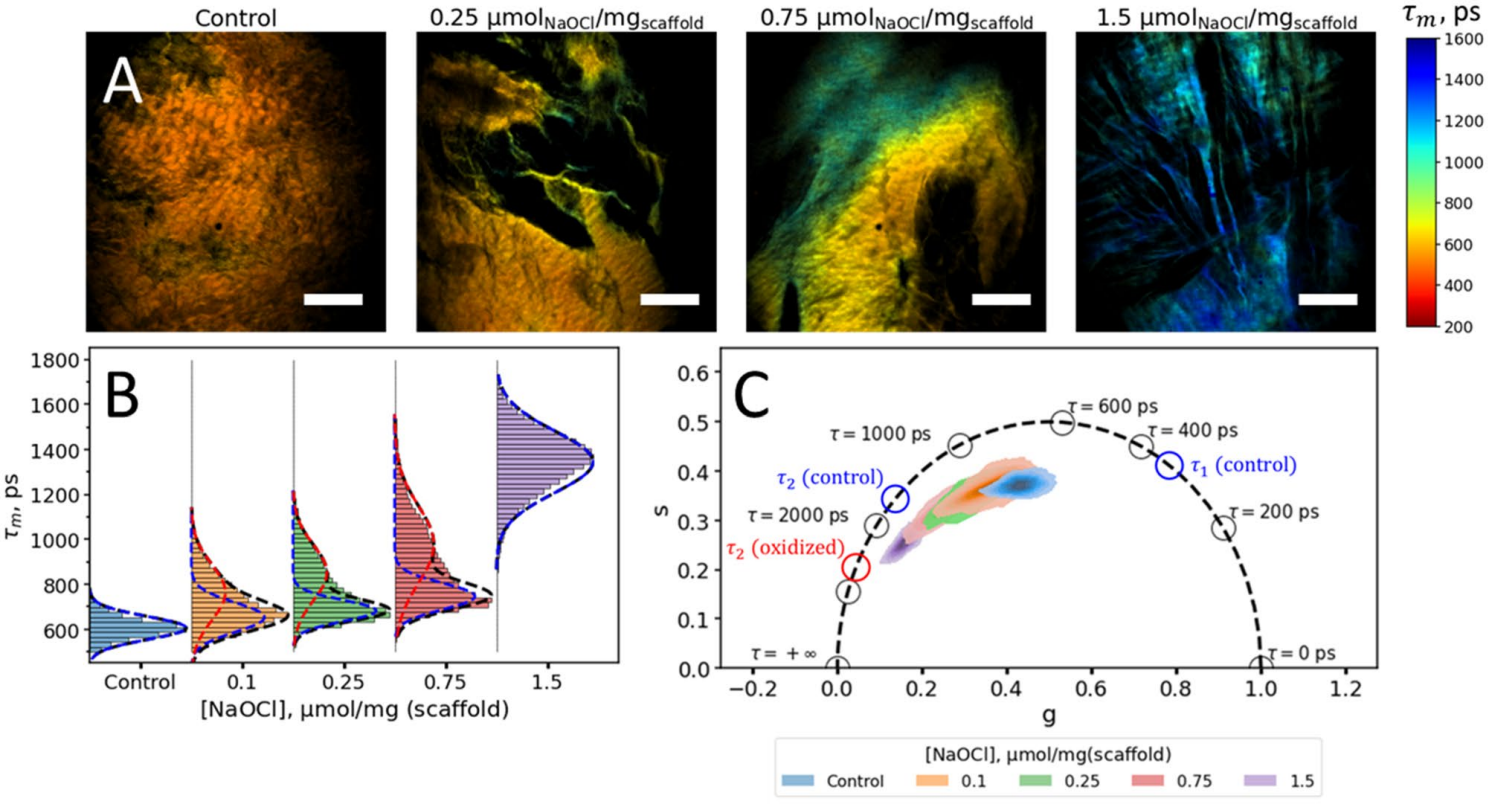
Fluorescence decay parameters of the decellularized bovine pericardium scaffolds cross-linked with genipin (DBP-G) treated with sodium hypochlorite. (**A**) Average fluorescence lifetime (τ_m_) maps of the control DPB-G scaffold and DPB-G scaffolds, treated with 0.25, 0.75, 1.5 µmol (NaOCl)/mg (scaffold). (**B**) Histogram distributions of the average fluorescence lifetime τ_m_ for DPB-G samples treated with different concentrations of sodium hypochlorite. The experimental fluorescence lifetime distributions were fitted by Gaussian distribution (control sample and sample treated with 1.5 μmol (NaOCl)/mg (scaffold)) or by a mixture of two Gaussian distributions. Blue and red dashed lines represent the respective components in the mixture, while black dashed lines represent the overall fit. (**C**) Phasor-plot of FLIM data for scaffolds treated with various concentrations of sodium hypochlorite. Blue and red circles indicate the fluorescence averaged decay lifetimes τ_1_ and τ_2_ of the control DBP-G sample and DBP-G treated with 1.5 μmol (NaOCl)/mg (scaffold) estimated using biexponential decay model averaged over all samples and all pixels. Fluorescence was excited at 800 nm and detected in the 350–680 nm range. Scale bars in panel (**A**) equal to 150 µm.

Figure 1A shows the maps of the average fluorescence lifetime τ_m_ for the control sample and samples treated with different concentrations of NaOCl. The fluorescence lifetime in control DPB-G scaffold (Fig. 1A) was homogeneously distributed over the field of view, while in the treated scaffolds, the values of average fluorescence lifetimes were heterogeneously distributed over the sample. Distribution histograms of the average fluorescence lifetimes are presented in Fig. 1B. For the control sample, the average fluorescence decay time had a narrow unimodal distribution with a median value of ~ 600 ps. For the treated samples, the distributions of the median values of the fluorescence lifetime were shifted to larger values of ~680, 740, 840 ps and ~1350 ps after exposure to 0.1, 0.25, 0.75 and 1.5 µmol NaOCl/mg scaffold, respectively.

The fluorescence lifetime τ_m_ in the samples treated with 0.1, 0.25 and 0.75 µmol NaOCl/mg scaffold had a pronounced bimodal distribution, likely corresponding to the signals from the oxidatively modified and non-modified areas of the biomaterial. This bimodality is also observed in several FLIM images of scaffolds processed with intermediate concentrations of hypochlorous acid. Specifically, for FLIM image of DBP-G scaffold treated with 0.75 µmol NaOCl/mg scaffold (Fig. 1A) we indicated that regions of DBP-G scaffolds both with homogeneous and heterogeneous fluorescence lifetime distributions were observed by FLIM. Additional examples of τ_m_ maps for DBP-G scaffolds treated with 0.1, 0.25 and 0.75 µmol (NaOCl)/mg (DBP-G) with visually homogeneous and heterogeneous distribution are shown in Fig. S2. To quantify the average fluorescence lifetime of these regions, the distributions of the fluorescence lifetime τ_m_ for the samples were fitted using a two-component Gaussian mixture model. The fluorescence lifetime of the cluster attributed to modified areas, which exhibited longer lifetimes (red dashed curves in Fig. 1B), was more sensitive to the NaOCl treatment, and its position changed from ~760 ps for 0.1 (NaOCl)/mg (scaffold) up to 850 and 980 ps for samples treated with 0.25 and 0.75 µmol NaOCl)/mg scaffold, respectively. The positions of the clusters with shorter lifetimes shifted mildly and were equal to ~ 650, 680 and 740 ps for the samples treated with 0.1, 0.25 and 0.75 µmol NaOCl/mg scaffold, respectively. The bimodal fit also allowed us to evaluate the fraction of the oxidized material in samples treated with different concentrations of sodium hypochlorite by calculating the fraction of pixels attributed to the cluster with a longer lifetime, which increased gradually with the increase of the hypochlorite concentration. The results of the analysis are summarized in Table 1.

**Table 1:** Fitting of the mean fluorescence lifetime distributions τ_m_ of the DPB-G scaffolds treated with NaOCl using Gaussian mixture model.

[NaOCl] μmol/ mg (scaffold)	τ_m_^(1)^, ps	σ^(1)^, ps	τ_m_^(2)^, ps	σ^(2)^, ps	Oxidized material fraction
0 (control sample)	605.4	50.7	–	–	0
0.1	653.1	65.0	763.9	123.6	0.35
0.25	682.3	47.3	851.6	123.7	0.45
0.75	746.4	58.1	976.8	172.4	0.53
1.5	–	–	1345.0	137.2	1.0

Parameters τ_m_^(1)^ and τ_m_^(2)^ represent the position of the maximum of the corresponding Gaussian curves, while σ^(1)^, σ^(2)^ represent the characteristic Gaussians’ widths. An oxidized material fraction was estimated as a fraction of pixels attributed to the cluster with a longer lifetime (i.e., cluster “2”).

After treatment with 1.5 µmol NaOCl/mg scaffold, the average fluorescence decay time had a maximum at ~ 1350 ps and its distribution was mono-modal, likely indicating that the majority of the sample underwent oxidative modification.

To better characterize the observed differences, we visualized the FLIM data using the phasor plot diagrams (Fig. 1C), in which each point represents fluorescence decay curves with a specific fluorescence decay time and changes in both fluorescence decay lifetimes and amplitude ratios are readily seen. In the case of mono-exponential decay, the corresponding point is located on the universal circle, while in the case of multi-exponential decay, the point is located inside the universal circle^30^. We observed that the distribution of both s and g phasor components shifts towards longer fluorescence decay lifetimes, which is caused by both changes in fluorescence lifetimes values (mainly longer fluorescence lifetime τ_2_) and the ratio between values of the fast and slow fluorescence decay components a_1_/a_2_. Distributions of the fluorescence decay components (amplitude of the short component a_1_ and fluorescence lifetimes τ_1_ and τ_2_) obtained using non-linear least-squares fitting are also presented in Supplementary Information (Fig. S3).

### FLIM assessment of DBP-G-scaffolds degradation induced by neutrophils

Upon scaffold implantation, an immediate response from damaged cells and tissues stimulates the migration of neutrophils to the site of injury and their activation^8^. There are several important features of the interactions between the damaged cells and neutrophils: (i) damaged cells release “alarm signals ”; (ii) plasma proteins attached onto a material trigger various immune signaling cascades to promote inflammation; (iii) activated platelets participate in the reactions of innate immunity. Because these conditions are not easily reproduced in in vitro experiments, we activated neutrophils by PMA, which stimulates the secretion of MPO^36^ and induces neutrophil extracellular traps (NETs) formation (in the presence of reactive oxygen species)^37^. In the presence of hydrogen peroxide, MPO synthesizes HOCl. In our experiments, we incubated DBP-G with neutrophils for 15–17 h (adding a new suspension of neutrophils four times). PMA was added 15 min after the beginning of each incubation, 100 µM of H_2_O_2_ was added in 1.5 h three times with 30 min intervals (see protocol in Sect. “Neutrophils isolation and incubation with scaffold”).

**Figure 2:**
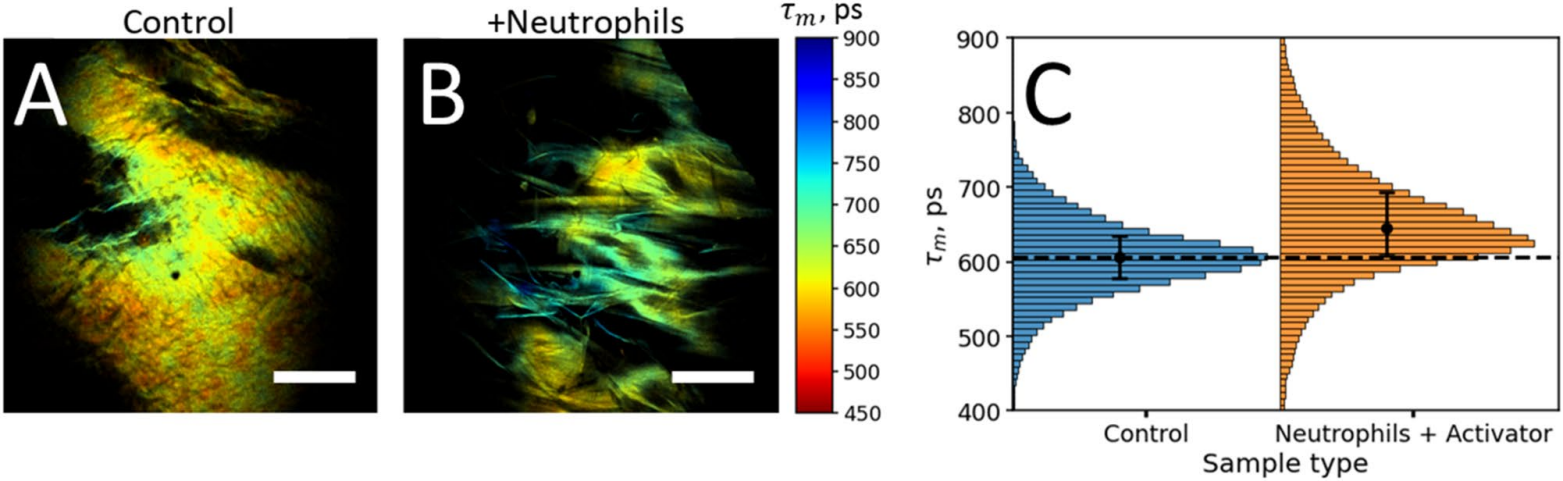
Fluorescence decay parameters of DBP-G scaffolds incubated with neutrophils. (**A**,**B**) Average fluorescence lifetime (τ_m_) maps of control DPB-G scaffold (**A**) and DPB-G scaffold incubated with neutrophils additionally activated with PMA (**B**). (**C**) Histogram distributions of the average fluorescence lifetime τ_m_ for control and treated DPB-G samples. Fluorescence was excited at 800 nm and detected in the 350–680 nm range. Scale bars in panels (**A**) and (**B**) are equal to 150 µm.

Given that MPT-FLIM was sensitive to the degradation of scaffolds in model experiments, we assessed the changes in fluorescence responses in DBP-G-scaffolds caused by exposure to the cells of the immune system-neutrophils. The representative average fluorescence lifetime maps of the control and DBP-G samples exposed to activated neutrophils are shown in Figs. 2A,B. The surface of the treated scaffold morphologically was more heterogeneous than the surface of the control sample. The neutrophils-treated samples also demonstrated the heterogeneous distribution of the average fluorescence lifetime over the sample, which was shifted towards longer lifetimes as compared to the control sample (Fig. 2A,B). The median value of the average fluorescence lifetime was shifted from 600 to 650 ps (*p* < 10^–4^), indicating a modification of the scaffold surface in the presence of the immune cells (Fig. 2C).

### Evaluation DBP scaffolds degradation using fluorescence of the HOCl-modified proteins

While cross-linked DPB-G displays a bright genipin-associated fluorescence, many other cross-links do not exhibit strong absorption and fluorescence in the visible range. Therefore, we further explored the possibility of assessing the degradation of DPB scaffolds with a cross-linker, which does not induce the formation of bright fluorescent cross-links. We attempted to perform FLIM measurements using bio-scaffolds that are cross-linked with the non-fluorescent agent-DBP scaffolds cross-linked with ethylene glycol diglycidyl ether (DBP-EGDE), which does not absorb or fluoresce in the visible range. The autofluorescence, in this case, may originate from the heterogeneous oxidation products of the molecular constituents of the scaffold (e.g., proteins), which have a low fluorescence yield but can be excited in the visible and even near-infrared spectral ranges^38^. The DBP-EGDE samples were treated with sodium hypochlorite. In water NaOCl hydrolyzes to hypochlorous acid-one of the major oxidizing agents released by activated neutrophils.

**Figure 3:**
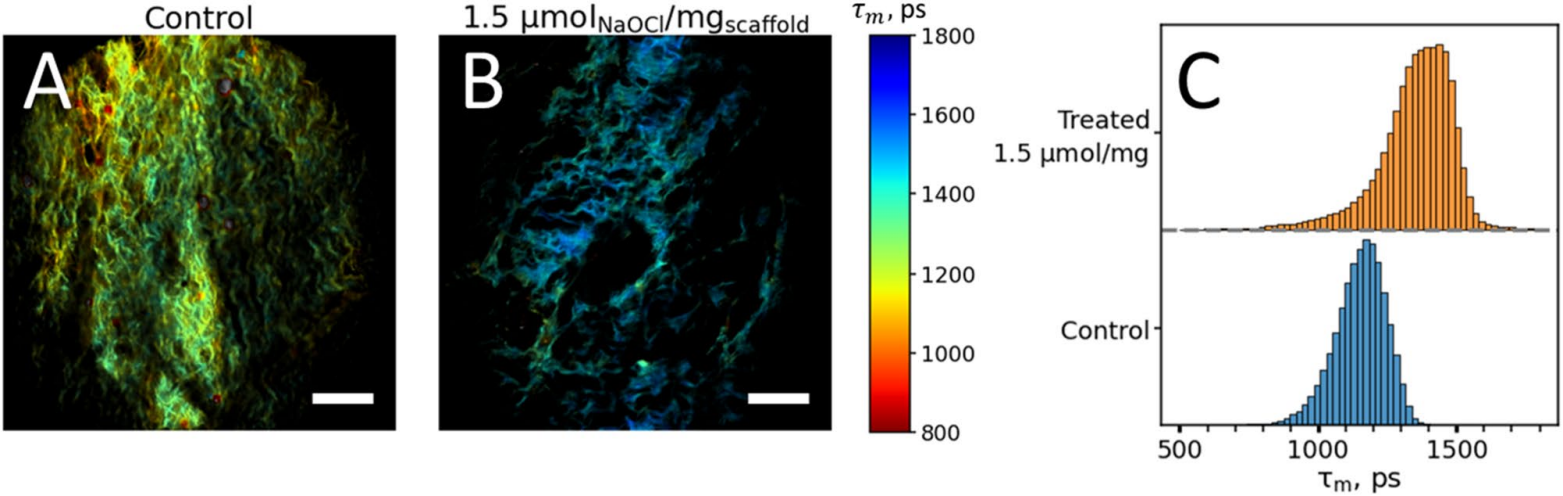
Fluorescence decay parameters of the decellularized bovine pericardium scaffolds cross-linked with ethylene glycol diglycidil ether and treated with sodium hypochlorite, modeling the immune response to the biomaterial. (**A**, **B**) Average fluorescence lifetime (τ_m_) maps of control DPB-EGDE scaffold (**A**) and DPB-EGDE scaffold, treated with 1.5 µmol (NaOCl)/mg (scaffold) (**B**). (**C**) Histogram distributions of the average fluorescence lifetime τ_m_ for DPB-EGDE samples treated with different concentrations of sodium hypochlorite. Fluorescence was excited at 730 nm and detected in the 350–680 nm range. Scale bars in panels (**A**) and (**B**) are equal to 150 µm.

The intensity of the fluorescence response from the oxidation products of proteins could be relatively low (quantum yield ~0.1–1%) with more effective excitation in the short-wavelength range^38^. We observed, that the fluorescence intensity of the DBP-EGDE scaffolds excited at 800 nm was ~100 fold lower than that of the DBP-G scaffolds, while DBP-EGDE optical response also exhibited strong second harmonic signal contribution to the optical response which hinders the fluorescence lifetime analysis (see details on the fluorescence intensity evaluation and second harmonic signal contribution in Supplementary Notes 1, 2). Therefore the fluorescence signal was excited at 730 nm and detected in the 350–680 nm range. The fluorescence intensity of the DBP-EGDE-scaffolds was ~100-fold lower than that of the DBP-G scaffolds. Yet, a distinct autofluorescence from the control and treated DPB-EGDE scaffolds was observed and fitted using the biexponential decay model (Fig. 3).

The maps of the average fluorescence lifetime τ_m_ for the control and treated DBP-EGDE scaffolds are shown in Fig. 3A,B. The morphology of these scaffolds differs significantly from that of DPB-G: namely, individual scaffold fibers were clearly observed. Secondly, the average fluorescence lifetime in the DBP-EGDE scaffolds was distributed heterogeneously both in the control and NaOCl-treated samples. This heterogeneity could be caused by the heterogeneous nature of the oxidation products, which presumably are the primary source of the observed fluorescence.

Figure 3C demonstrates the distribution histograms of the average fluorescence lifetime τ_m_. The τ_m_ values in the sample treated with hypochlorite increased compared to the control sample from ~ 1160 ps to ~ 1360 ps. Thus, in DBP-EGDE scaffolds, where the main source of autofluorescence is most likely the oxidation products of scaffolds ’ proteins, the fluorescence decay parameters assessed via MPT-FLIM can be used to evaluate the degradation of scaffold caused by oxidation.

### Changes in the fluorescence decay parameters correlate with micromechanical properties of DPB-scaffolds

**Figure 4:**
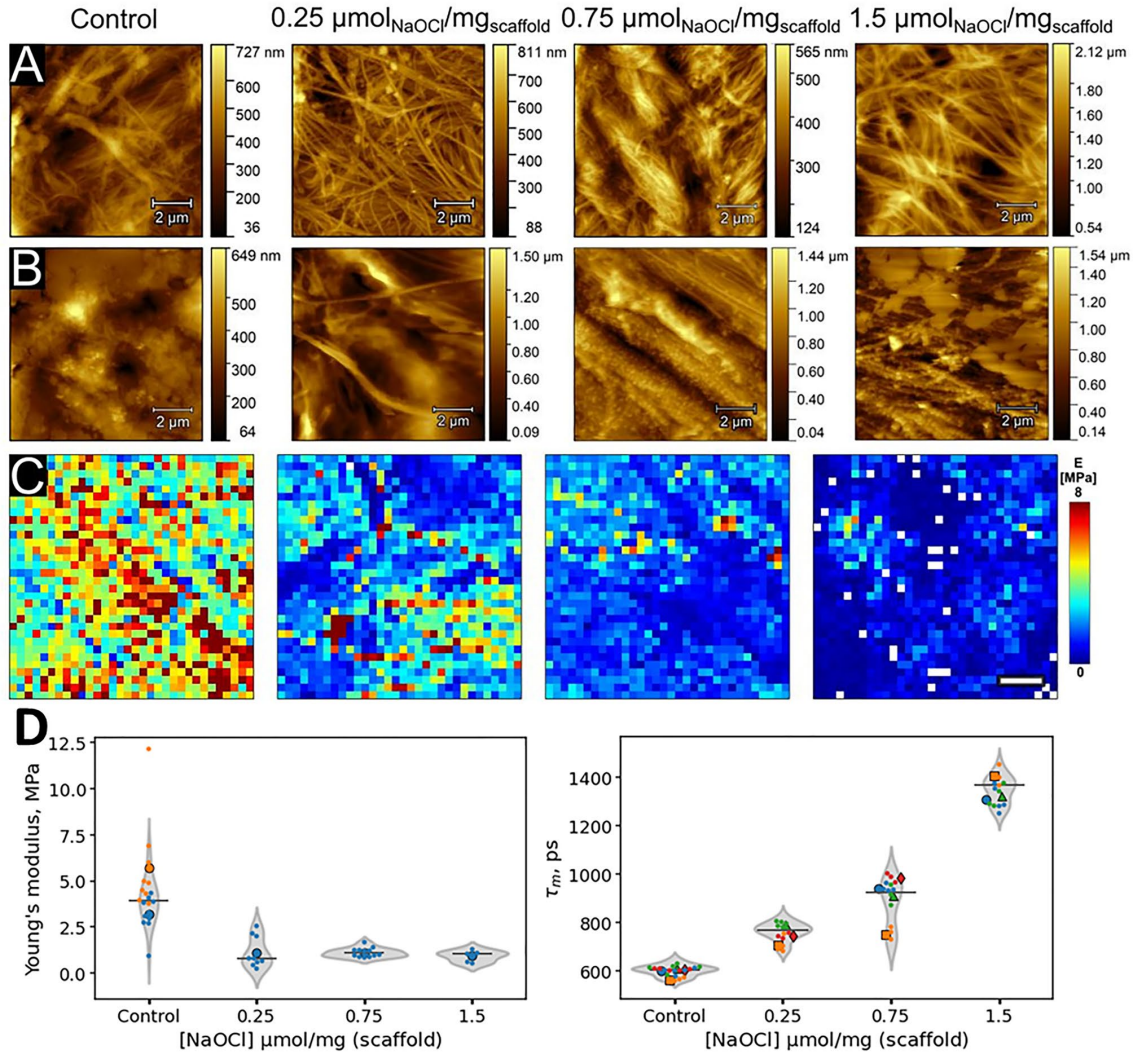
Representative AFM data obtained on the decellularized bovine pericardium scaffolds cross-linked with genipin (DBP-G) treated with sodium hypochlorite. (**A**–**B**) Topography images of the control DPB-G scaffold and DPB-G scaffolds, treated with 0.25, 0.75, 1.5 µmol (NaOCl)/mg (scaffold): fibrillar areas (**A**) and amorphous areas (**B**). (**C**) Nanomechanical maps (distribution of Young’s modulus, *E*) of the control and treated DBP-G samples. All images have the same color-coded scale for the modulus; the scale bar is 2 µm. (**D**) Young’s modulus of the control DPB-G sample and DBP-G samples treated with 0.25, 0.75 and 1.5 µmol (NaOCl)/mg (scaffold) (left panel), and increase in the fluorescence lifetime $\tau_{m}$ for the same samples (right panel). Data in panel (**D**) is represented in the form of superplot: small dots represent the average Young’s modulus and the fluorescence lifetime τ_m_ averaged over the one image, while large dots represent the average over the replicated experiments. Horizontal lines represent the total average obtained for treated and untreated DBP-G scaffolds.

An independent non-optical method was additionally used to document the modification of the scaffold by NaOCl. We utilized AFM for the characterization of the changes in micromechanical properties of DPB-G samples treated with NaOCl. The control samples were quite heterogeneous in terms of Young’s modulus with typical values in the range of 5–20 MPa (Fig. 4A,B). After treatment with NaOCl, we observed a decrease in Young’s modulus of the treated DPB-G-samples relative to the control samples. This decrease was proportional to the used NaOCl concentration (Fig. 4C,D). The lowest decrease (2.7-fold) was detected for 0.25 µmol (NaOCl)/mg (scaffold), and the highest decrease (5.5-fold) for 1.5 µmol (NaOCl)/mg (scaffold). The “softening” of treated DPB-G samples can be related to the degradation of individual scaffold fibers and cross-linking between them. However, there were no clear differences between the topographical images of control and treated samples, all of which were quite heterogeneous and contained both fibrillar (Fig. 4A) and amorphous areas (Fig. 4B). We also found that the changes in the mechanical properties correlated with the average fluorescence lifetime τ_m_ for the same samples, demonstrating that the fluorescence decay parameters could be used as indicators of the changes in the mechanical properties (Fig. 4D).

## Discussion

Accurate and fast characterization of the physicochemical properties of the engineered and natural tissues is of paramount importance for modern regenerative medicine. Optical techniques, in particular, MPT-FLIM, provide the ability to assess the state of cells and tissues with a subcellular resolution by mapping the parameters of the fluorescence decay curves. The fluorescence decay parameters of the endogenous fluorophores, such as NAD(P)H and FAD, allow evaluations of the metabolic status of cells and tissues^17^, the simultaneous use of cells’ morphology and their fluorescence decay parameters allows one to distinguish between different types of cells both in vitro and in vivo using FLIM^39,40^. The use of the multiphoton excitation in the near-infrared range permits to scan relatively deep tissue layers, hence to localize and characterize structural proteins, to determine the parameters of the microcapillaries and study physiological processes in vivo^27,41^. FLIM has also been used in the assessment of the properties of the engineered biomaterials^18–23^. In this work, we applied FLIM to assess the modification of collagen scaffolds in model systems mimicking the pro-inflammatory response of the immune cells.

Tissue damage and foreign material implantation induce an acute inflammatory response. The major immune cells recruited to the implantation site are phagocytes-neutrophils and macrophages in the pro-inflammatory M1 state. Infiltration of scaffolds by neutrophils occurs during the first week after the implantation of materials into experimental animals^42,43^. The appearance of neutrophils and their replacement by macrophages associated with the scaffold degradation have been well documented for a number of materials, including cross-linked collagen-based biomeshes^21,44^. However, oxidative modification of materials by activated phagocytes has not been sufficiently studied.

Activated phagocytes generate ROS-hydroxyl and hydroperoxyl radicals (HO· and HOO·), peroxynitrite (ONOO-), and hypochlorous acid (HOCl ↔ H^+^  + OCl^−^)-to fight bacterial and viral pathogens as well as other foreign intruders at the site of damage. HOCl is a powerful oxidant produced by neutrophil myeloperoxidase in the presence of H_2_O_2_ and Cl^-^. HOCl is apparently more effective in oxidizing and degrading carbonaceous nanomaterials such as carbon nanotubes and graphene oxide than other ROS species^12,45,46^.

We hypothesized that a potent oxidizing agent HOCl (redox potential of the couple HOCl/Cl^−^, H_2_O is ~1.3 V^10^) can oxidatively modify and biodegrade biomaterials, including pericardium scaffolds cross-linked with genipin and ethylene glycol diglycidyl ether. At the site of inflammation in vivo, activated neutrophils continuously produce HOCl, and new cells replace apoptotic neutrophils. In our in vitro experiments, we repeatedly added a new aliquote of NaOCl. The number of additions and the amount of NaOCl added depended on the weight of the scaffold and on the desired reagent concentration. NaOCl concentrations used in our experiments were comparable to their expected amounts in acute inflammation. The local steady-state concentrations of HOCl at the inflammatory site may exceed 100 μM, ensuring efficient oxidation of foreign material^47^. In our experiments, the concentration of 0.1 µmol NaOCl per mg of the pericardium was achieved by a single addition of 300 µM of NaOCl, whereas the concentration of 0.25 µmol NaOCl per mg of the scaffold was achieved with two additions: 450 µM and 300 µM of NaOCl were added with 15 h interval.

The fluorescence lifetime of a large number of fluorophores is sensitive to the pH of the fluorophore ’s environment. The observed changes in the fluorescence lifetime could be related to the biochemical processes occurring in the cells and tissues and/or to changes in the properties of single fluorophores. For example, it was found that the endogenous fluorescence of NADH in cells was pH-sensitive^48^. Based on this, pH in a single cell can be determined by fluorescence lifetime imaging of NADH. Of note, the magnitude of pH-induced changes was larger in the nuclei than in other intracellular areas (∆τ_m_ decrease was 200 ps if ∆pH ~ 1.0 in the range of pH from 5.0 to 10.0). Design and synthesis of NIR fluorescence lifetime pH-sensitive fluorophores for intracellular and extracellular pH measurements were described in a number of studies^49,50^. Genetically encoded pH sensor showed the highest sensitivity in the pH range from 7.5 to 8.0 when the increase in fluorescence lifetime was about 450 ps^50^. pH-sensitive fluorescence lifetime probes were developed to demonstrate the potential of FLIM to quickly determine the acidity of a region in vivo^51^. Given the pH sensitivity of FLIM, we monitored pH of our samples because the stock solution of NaOCl is highly basic. Samples of DBP were placed into (50 mM NaH_2_PO_4_ in PBS, pH 7.3–7.4). After all additions into the sample (1.5 µmol NaOCl per mg DBP-G), pH shift did not exceed 0.15 units, and bleaching of the material was observed. Of note, the pH changes occur quickly after the addition of a micro-aliquot of NaOH into the solution and vortexing, while bleaching of the treated samples and subsequent chemical/biochemical changes occurred on a longer time scales of few days (Fig. S4).

We employed FLIM to study the oxidative modification of the pericardium scaffold and found that changes in FLIM responses were highly sensitive to modifications of cross-linked with genipin scaffold to treatment with NaOCl. The lifetime of the control samples not exposed to the oxidant was the shortest (τ_m_ ~600 ps), and was distributed unimodally (Fig. 1B). NaOCl caused the increase in fluorescence lifetime and the changes of lifetime distribution. At relatively low biologically relevant concentrations of NaOCl, (0.1 and 0.25 µmol per mg of scaffold), a bimodal distribution of fluorescence lifetime was observed. This bimodality could be due to a decrease in the proportion of native material and the appearance of oxidized DBP-G. We assumed that the cluster with a shorter fluorescence lifetime (blue lines in Fig. 1B) corresponded to the superposition of the native and slightly oxidized scaffold, while the clusters with a longer lifetime (red lines) could be attributed to the material with a higher degree of oxidation. This approach allowed us to obtain and quantitatively characterize DBP-G oxidation with hypochlorite (Table 1). With the increase of the amount of added oxidizing agent (0.1 < 0.25 < 0.75 µmol NaOCl/mg scaffold), the maxima of both τ_m_ distribution curves dose-dependently shifted towards higher values of τ_m_, and at the same time, the contribution of the higher oxidized form of the material to the total fluorescence lifetime increased. At 1.5 µmol NaOCl/mg scaffold, completely oxidized DBP-G was again characterized by mono-modal τ_m_ distribution but with the maximum at 1.35 ns. The non-Gaussian shape of the distribution may be accounted for the material damage upon strong oxidation.

The observed change of the fluorescence lifetime was accompanied by alterations of the scaffolds ’ mechanical properties, as revealed by AFM, Fig. 4. At NaOCl concentration of 0.25 µmol per mg scaffold, Young’s modulus of the DBP-G decreased almost threefold. Further increase in the concentration of hypochlorite did not cause significant changes in the elastic properties of the pericardium surface. It can be assumed that low concentrations of hypochlorite (about 0.25 μmol per mg) are sufficient to modify all surface groups of the scaffold that are prompt to oxidation. A further increase in NaOCl concentration results in the modification of deeper layers of the material, which was tested by FLIM, by both NaOCl and long-lived radical intermediates formed because of protein oxidation. We note that correlation of fluorescence intensity ratio and average fluorescence lifetime with macroscopic Young’s Modulus measured by tensile testing was also observed for DBP in previous works^20^.

We also attempted to assess the sodium hypochlorite-induced oxidative modification of the scaffolds cross-linked with EGDE. The intensity of the fluorescence signal, in this case, was significantly lower in comparison with that of the DPB-G scaffolds, likely due to very low quantum yields of the oxidation products^38^. The average fluorescence lifetime was higher than for genipin (600 ps vs. 1.16 ns) for control samples, and only a slight increase of the τ_m_ was observed upon hypochlorite treatment.

Neutrophil MPO is the only enzyme capable of generating physiologically significant amounts of HOCl. During neutrophil activation at the site of tissue damage, the neutrophil granules release their contents into the phagosomes and extracellular space, including MPO and digestive proteins like collagenase and gelatinase. Bulky enzymes cannot penetrate into a scaffold, thus the degradation by digestive enzyme is a surface erosion process^52^. To the best of our knowledge, MPO-induced oxidative degradation of biomaterials was not considered in previous studies. Nevertheless, MPO-generated free radicals and oxidants can migrate into surface layer of the material to induce near-surface degradation.

MPO comprises about 5% of the dry mass of the neutrophil and is released into the phagosome up to a concentration of about 1 mM^53,54^. Additionally, MPO is one of the key components of the ‘sticky network’-neutrophil extracellular traps^55^. One can expect very high concentration of MPO at inflammatory site in the close proximity to the material surface. At the same time, NADPH oxidase is assembled on the plasma membrane and generates superoxide radicals which dismutate to form H_2_O_2_^56^. In inflamed tissue, steady state concentrations of hydrogen peroxide in extracellular medium can be as high as 100 µM^57^. Hydrogen peroxide feeds the MPO catalytic cycle enabling the enzyme to oxidize chloride ions and produce HOCl (second order rate constant is 2.5 × 10^4^ M^−1^ s^−1^ ^53^). HOCl is a powerful oxidant that non-specifically attacks not only foreign materials but also neutrophils themselves, thus causing their apoptotic death. During the acute inflammatory response, the replacement of apoptotic neutrophils by the new ones ensures the presence of activated neutrophils near the scaffold for 5–7 days. This long-term presence of activated neutrophils and NETs in the scaffold area results in massive production of HOCl by MPO. The in vivo MPO-depended degradation of highly resistant structures such as carbon nanotubes has been documented^58^.

In a separate study we demonstrated that DBP-G scaffolds activated neutrophils in whole blood and cause ROS generation, MPO secretion and NETs formation (unpublished results).

As the final step, modifications of scaffolds directly by incubation with activated neutrophils were assessed. In our experiments, we renewed the neutrophil suspension added to the scaffold four times to ensure the prolonged co-incubation of the scaffolds with activated cells (the total incubation time was 15–17 h). In 60–90 min periods of time after the beginning of each incubation, when the PMA-activated cells were dying^49^, we were adding H_2_O_2_ (100 µM × 3 times) to provide MPO with a source of oxidizing equivalents needed for HOCl synthesis. We found that for DBP-G scaffolds incubated with activated cells a small yet statistically significant increase of the median value of the average fluorescence decay time τ_m_ from 600 ps for the control sample to ~ 650 ps for the treated sample was observed. The fluorescence decay time τ_m_ in the scaffold sample incubated with neutrophils was also more heterogeneously distributed over the sample, demonstrating the fibrillary regions with the increased fluorescence lifetime τ_m_ (Fig. 2B). As compared to the results obtained for experiments with NaOCl-induced oxidation, the results obtained for experiments with scaffolds incubated with neutrophils are close to the effects observed for the concentration of 0.1 μmol (NaOCl)/mg (scaffold), where a median value of the fluorescence decay time was equal to ~680 ps.

In our experiments, the total incubation time of DBP-G with activated neutrophils was 15–17 h, whereas implanted materials are exposed to neutrophils for several days. After subcutaneous implantation of poly[(L-lactide)-co(ε-caprolactone)] scaffolds in animals, the injected material was infiltrated with neutrophils for a week after surgery^42^. Due to high steady-state concentration of HOCl (about 100 µM^47^) at inflammatory site one can expect significant scaffold modification. Using attenuated total reflectance Fourier transform infrared spectroscopy, Sutherland et al. have shown that in vivo degradation of polyetherurethane elastomers may involve both hypochlorous acid and nitric oxide-based oxidants^59^. Interestingly, HOCl and peroxinitrite appear to attack polyetherurethane at different sites. Our study demonstrates that FLIM is a useful tool for the evaluation of extracellular matrix modifications by oxidative stress generated by immune cell.

## Conclusion

Modification of tissue-engineered constructs occurring early after the implantation is essential since it affects the immune response and wound healing. Here, we showed that oxidative modifications of the collagen-based (decellularized pericardium) scaffolds by hypochlorous acid can be quantitatively characterized using MPT-FLIM. We demonstrated that the oxidation processes can be quantified using the changes of the fluorescence lifetime of either fluorescent cross-links (e.g., genipin) or by changes in the fluorescent response of collagen oxidation products. In experiments with DBP-G oxidation by sodium hypochlorite, changes in the modality of fluorescence lifetime distribution correlated with different degrees of oxidation and changes in the mechanical properties assessed by AFM. We also showed that a less intense fluorescence stemming from the scaffold collagen oxidation products can also be assessed by FLIM. We conclude that the FLIM protocol can be used for rapid quantitative label-free assessment of the properties of biomaterials.

## Supplementary Information

Supplementary Figures.
