# Supplementary material for: Detection of HOCl-driven degradation of the pericardium scaffolds by label-free multiphoton fluorescence lifetime imaging
Source: Sci Rep. 2022 Jun 20;12:10329. doi: 10.1038/s41598-022-14138-5 (PMC9209456; doi:10.1038/s41598-022-14138-5)
Supplement: Supplementary file 1 — Supplementary Figures. [file 41598_2022_14138_MOESM1_ESM.docx]

## *Supplementary information*

Detection of HOCl-driven degradation of the pericardium scaffolds by label-free multiphoton fluorescence lifetime imaging

Yakimov B.P.^1,2,#^, Vlasova I.I.^1,3,#^, Efremov Y.M.^1,3^, Maksimov E.G.^4^, Shirshin E.A.^2,1,^*, Kagan V.E.^3,5^, Timashev P.S.^1,3,6^*

^1^World-Class Research Center "Digital biodesign and personalized healthcare", Sechenov First Moscow State Medical University, Trubetskaya 8, Moscow, 119048, Russia

^2^Faculty of Рhysics, M.V. Lomonosov Moscow State University, 1-2 Leninskie Gory, Moscow, 119991, Russia

^3^Department of Advanced Biomaterials, Institute for Regenerative Medicine, Sechenov First Moscow State Medical University, Trubetskaya 8, Moscow, 119048, Russia

^4^Faculty of Biology, M.V. Lomonosov Moscow State University. 1-12 Leninskie Gory, Moscow, 119991, Russia

^5^Center for Free Radical and Antioxidant Health, Department of Environmental and Occupational Health, University of Pittsburgh, Pittsburgh, PA 15261 USA

^6^Faculty of Chemistry, M.V. Lomonosov Moscow State University, 1-3 Leninskie Gory, Moscow, 119991, Russia

# – both authors contributed equally to this work

*E.S.: [shirshin@lid.phys.msu.ru](mailto:shirshin@lid.phys.msu.ru) , P. T.: [timashev_p_s@staff.sechenov.ru](mailto:timashev_p_s@staff.sechenov.ru)

**Supplementary Note 1. On the evaluation of the second harmonic contribution to the detected decay curves**

The spectral characteristics of the FLIM detection system used in the experiments do not allow one to exclude the contribution of the second harmonic (SH) signal to the optical response from the investigated samples. Thus, the contribution of the SH signal should be properly evaluated.

In our experiments, we found out that the second harmonic signal was 1-2 orders of magnitude lower than the fluorescent response observed for the DBP-G samples excited at 800 nm and DBP-EGDE samples excited at 730 nm, while the characteristic ultrafast SH response was observed only for the DBP-EGDE samples excited at 800 nm. In Figure S1 we showed intensity decay curves obtained as an integral over the FLIM images of DBP-EGDE samples (both control and treated with 1.5 µmol NaOCl/mg scaffold) excited at 730 and 800 nm and the decay curve of control (untreated) DBP-G sample excited at 800 nm for comparison. The decay curves of the DBP-EGDE samples (both oxidized and untreated) excited at 800 nm demonstrated the contribution of the SH signal, which manifests itself in the optical decay curve as an ultrafast response with a characteristic decay time of ~100 ps (which is comparable to the instrument response function). At the same time, decay curves of the optical responses of DBP-G (excitation at 800 nm) and DBP-EGDE (excitation at 730 nm) demonstrated characteristic decay lifetimes of the fast decay component significantly larger than the instrument response function – nearly 380 ps for the DBP-G control scaffold and 450 ps for the DBP-EGDE scaffolds. Figure S1B shows fluorescence decay curves normalized to the intensity maximum, demonstrating the ultrafast component corresponding to the second harmonic signal in the DBP-EGDE sample excited at 800 nm. Thus, we assume that the influence of the second harmonic signal, although not completely excluded by the spectral characteristics of the FLIM detection system, is not significant in the experiments with DBP-G samples excited at 800 nm and DBP-EGDE samples excited at 730 nm, as the ultrafast decay component is not detected.

*
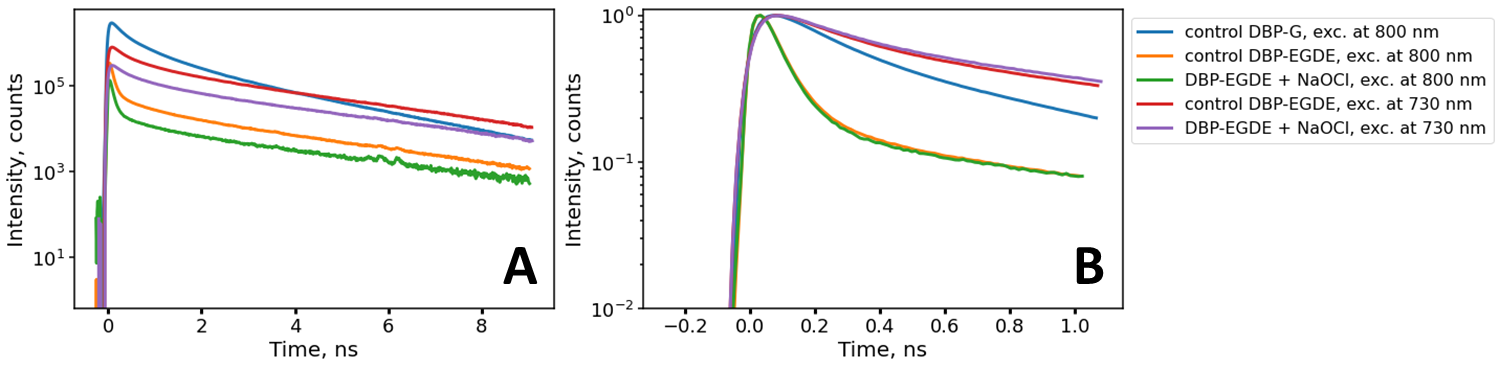
*

**Figure S1.** Raw (A) and normalized to the intensity maximum (B) integral fluorescence decay curves obtained for DBP-G and DBP-EGDE untreated and treated with 1.5 µmol (NaOCl)/mg scaffold obtained with excitation at 730 and 800 nm. The contribution of the second harmonic signal to the overall optical signal was observed as an ultrafast decay component with a characteristic lifetime of ~100 ps in the DBP-EGDE scaffolds with an excitation at 800 nm.

**Supplementary Note 2. On the estimation comparison of DBP-G and DBP-EGDE samples intensity.**

The fluorescence decay curves presented in Fig. S1 were used to evaluate the fluorescence intensity of the samples under the same scanning parameters. Specifically, we compared the fluorescence intensity integrated within the time range from 0.3 to 12 ns for the DBP-G and DBP-EGDE samples excited at 800 nm obtained with identical scanning parameters. The time range from 0 to 0.3 ns was excluded due to the contribution of the second harmonic signal in DBP-EGDE samples at this excitation wavelength. We obtained that the fluorescence intensities of the DBP-EGDE samples were equal to ~2.6% and ~1.1% of the fluorescence integral intensity of the DBP-G control sample for the control DBP-EGDE sample and DBP-EGDE scaffold treated with 1.5 µmol (NaOCl)/mg scaffold, respectively. Low fluorescence intensity response of DBP-EGDE scaffolds at 800 nm could be caused both by the low concentration of collagen fiber oxidation products that absorb fluorescence at a given wavelength or low quantum yield of the fluorescence products compared to the fluorescent cross-links in the DBP-G.

*
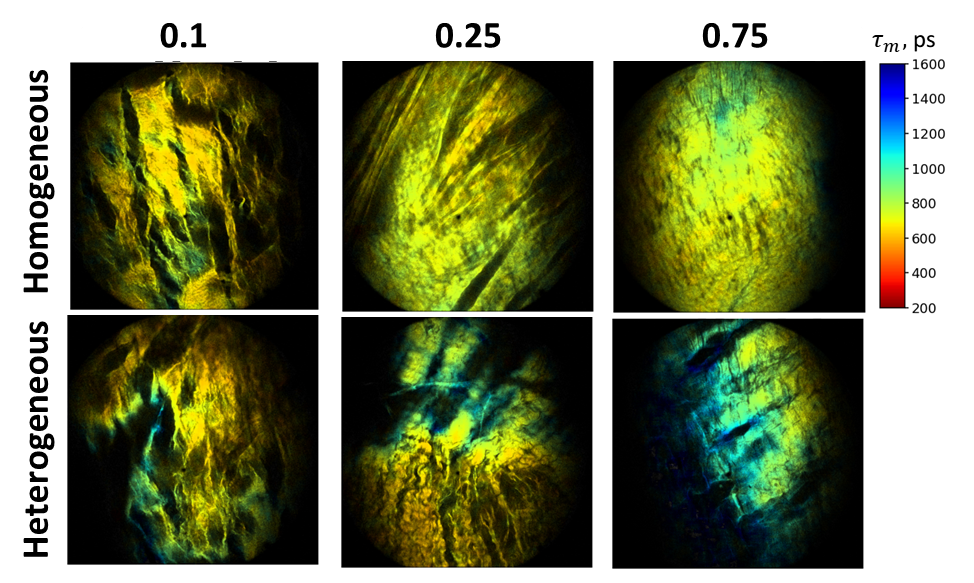
*

**Figure S2.** Average fluorescence lifetime (τ_m_) maps of the DPB-G scaffold treated with 0.1, 0.25 and 0.75 µmol (NaOCl)/mg (scaffold) illustrating visually homogeneous and heterogeneous distribution of the average fluorescence lifetime τ_m_


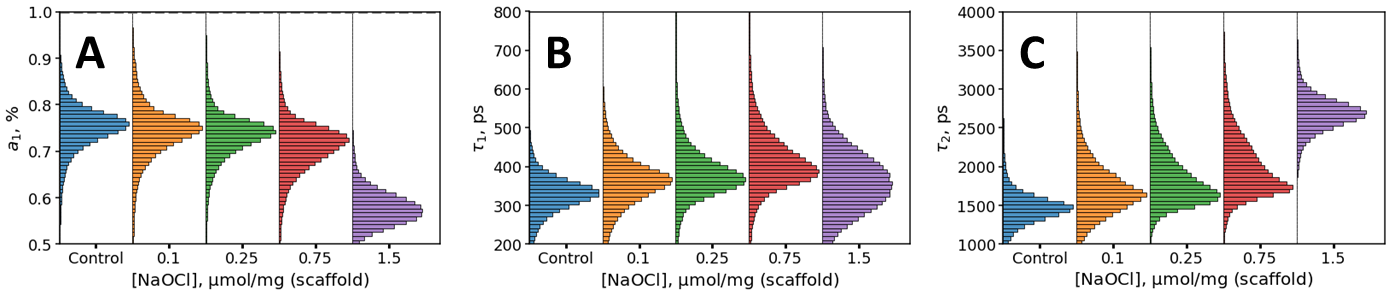


**Figure S3.** Histogram distributions of the amplitude a_1_ of the fast decay component (A) and fluorescence lifetimes τ_1_ (A) and τ_2_ (B) for DPB-G samples treated with different concentrations of sodium hypochlorite.

**Figure S4.** Samples of DBP-G after NaOCl treatment. Bleaching of DBPG samples accompanies the oxidation of scaffolds by hypochlorous acid.

Micro-aliqoutes of 0.5-1 µl NaOCl (1.7 М) were added to the scaffolds placed into 4 ml of buffer (PBS+50mM NaH_2_PO_4_, pH 7.3-7.4). NaOCl was added once or twice a day for 5-10 days up to the final concentration indicated in the Figure. Total incubation time was 12 days.

Control sample was prepared three days before measurement and kept at 4C.
